# Supplementary material for: Effect of storage temperature on the dispersibility of commercially available 0.1% fluorometholone ophthalmic suspension
Source: PLoS One. 2022 Nov 3;17(11):e0277311. doi: 10.1371/journal.pone.0277311 (PMC9632795; doi:10.1371/journal.pone.0277311)
Supplement: Attachment — Submitted filename: Response_to_Reviewers_TO_final20221018.docx [file pone.0277311.s001.docx]

***Response to Reviewer 1’s Comments***

We would like to thank #Reviewer 1 for the valuable suggestions on our manuscript. We have carefully read all of your comments and suggestions and have made the corrections in the revised version of the manuscript. Detailed responses to your comments are listed below, and we have highlighted all changes which can be viewed as tracked changes in the file labeled “Revised Manuscript with Track Changes.” We hope that the revised manuscript would be satisfactory for publication in *PLoS ONE*.

***Q1. In all manuscript, section numbers should be checked. The use of section numbers in only subsections is inappropriate.***

A1. According to #Reviewer 1’s comment, we removed the numbering in the Results section (Lines 188–189, 200–201, 223–234).

***Q2. Lines 91-93, etc.: In all manuscript and in references, the use of capital letters should be checked.***

A2. The reviewer’s comments are very important. We checked the use of capital letters in this manuscript. The name of products and vendors are not changed, since these are original notation. Thank you for pointing out this (Lines 91–93).

**Q*3. Line 111: “Further,” should be replaced by “Furthermore,”.***

A3. Thank you very much for pointing this out. We revised to “Furthermore” from “Further” in Materials and Methods (Lines 111).

***Q4. Lines 120, 131, 146-147, etc.: In all manuscript, there is no coherence in the use of capital and small letters during the spelling of subsection titles.***

A4. The reviewer’s comment is correct. We unified the use of capital and small letters in the spelling of subsection titles in Materials and Methods and Results. Please refer to the comments in the file labeled “Revised Manuscript with Track Changes” (Lines 103,125,140,149,172,181,188-189).

***Q5. Lines 162, 164, 165, 170, etc.: In all manuscript, please use subscript for “s” in “Vs”, for “p” in “Dp”, for “g” in “ρg” and for “f” in “ρf”.***

A5. Thank you for pointing out this. We have incorporated subscript “s”, “p”, “g”, and “f” into Stokes’ equation (Lines 32–33, 155, 157-158, 163, 228–229, Table 2).

***Q6.*** ***Lines 218, 251, etc.: In all manuscript, please use small letters in the spelling of “Low” and “Room”.***

A6. In order to respond to the reviewer’s comment, we replaced “Low” and “Room” with “low” and “room” throughout the manuscript. Please refer to the comments in the file labeled “Revised Manuscript with Track Changes” (Lines 446, 455, 464, 474).

***Q7. Lines 222-223; 255-256; 275-278; 300-301; etc.: The place of these statements is inappropriate!!! They should be given in figure captions, not in text.***

A7. The reviewer’s comments are very important. In order to respond to the reviewer’s comment, we moved all Figure Captions after the References (Lines 429–488).

***Q8. Line 418: Please add a “Conclusion” section.***

A8. Thank you for pointing out this. We added the Conclusion section (Lines 345–359).

***Q9.*** ***Line 419: “Reference” should be replaced by “References”.***

A9. According to #Reviewer 1’s comment, we corrected to “ References” (Line 361).

***Q10.*** ***Lines 419-492: This section should be checked according to the reference format of plos One.***

A10. The reviewer’s comment is correct. We revised the reference format according to the submission guidelines of PLoS ONE (References, Lines 361–426).

***Q11.*** ***Figure captions should be presented before figures.***

A11. Thank you very much for pointing this out. In order to respond to the reviewer’s comment, we moved all Figure Captions after the References (Lines 429–488).

***Q12.*** ***Fig. 3: The quality of Fig. 3 should be improved.***

A12. In order to respond to the reviewer’s comment, we improved the quality of Figure 3 (Figure 3).

***Q13.*** ***Fig. 5:• “frequency” should be replaced by “Frequency”. • In x-axes, “Particle Size” should be replaced by “Particle size”.***

A13. The reviewer’s comment is correct. We revised “frequency” to “Frequency”, and “Particle Size” to “Particle size” in Figure 5 (Figure 5).

Thank you for great comments.

***Response to Reviewer 2’s Comments***

We would like to thank #Reviewer 2 for the valuable suggestions on our manuscript. We have carefully read all of your comments and suggestions and have made the corrections in the revised version of the manuscript. Detailed responses to your comments are listed below, and we have highlighted all changes which can be viewed as tracked changes in the file labeled ‘Revised Manuscript with Track Changes’. We hope that the revised manuscript would be satisfactory for publication in *PLoS ONE*.

***Q1. The manuscript, although interesting and well-designed and conducted, needs extensive language editing. Some sentences are exceedingly long and difficult to follow.***

A1. In order to respond to the reviewer’s comment, this manuscript has been re-edited by English editing service Editage (Job code: BCIO_1_3).

***Q2. I suggest the authors to omit numbering in the results section and use plain subheading; or alternatively without any subheadings. If subheadings are preferred a similar style may be useful in the Discussion section as well.***

A2. Thank you very much for pointing this out. We removed the numbering in the Results section, and applyied the plain subheadings (Lines 187–234).

Thank you for great comments.

***Response to Reviewer 3’s Comments***

We would like to thank #Reviewer 3 for the valuable suggestions on our manuscript. We have carefully read all of your comments and suggestions and have made the corrections in the revised version of the manuscript. Detailed responses to your comments are listed below, and we have highlighted all changes which can be viewed as tracked changes in the file labeled ‘Revised Manuscript with Track Changes’. We hope that the revised manuscript would be satisfactory for publication in *PLoS ONE*.

***Q1. It is however a very technical manuscript which is not so easy to read for the clinician. This is partly caused by the often long sentences with significant sub-sentences. I have no questions concerning the content. The research is soundly executed, and is presented and discussed step by step. I do recommend shortening of the often complex and long sentences to increase the readability of this technical manuscript. Furthermore a suggestion to perhaps incorporate advices for the*** ***ophthalmic clinician, i.e. apart from the frequency of administration, a storage or use advice may also be discussed.***

A1. According to #Reviewer 3’s comment, this manuscript has been re-edited by English editing service Editage (Job code: BCIO_1_3). We also incorporated the Conclusion section including the recommendations for ophthalmic clinicians and pharmacists (Lines 345–359).

Thank you for great comments.
